# Practical Notes and Formulæ

**Published:** 1879-02-20

**Authors:** 


### PRACTICAL NOTES AND FORMULÆ.

**After-Pains.**—I have not given a dose of opium for this purpose for ten years, and find that I get along much better without than with it. In the olden time, I prescribed five-grain doses of diaphoretic powder, and usually had milk fever, loss of appetite and constipation to look after. Now, the prescription in the ordinary case is:

℞ Tincture aconite.........................gtt. v.
Tincture mycrotys...................gtt. xx.
Water....................................℥ iv.

A teaspoonful every one, two or three hours, as needed. Strange to say, there is no fever, irritation or other unpleasantness; and though the pain is not at once arrested, it is mitigated, and after a reasonable time stops. Involution of the uterus goes on better, and I think with a better recovery. In some cases the viburnum will be found the better remedy, and in others, where there is also abdominal pains, nux may be occasionally required. I think a trial of these methods will give satisfaction.—*Eclectic Medical Journal.*

[Dr. H. E. Hurst, of Alabama, says that he has found citric acid in five-grain doses every two to three hours to be an efficient remedy in after-pains. The *modus operandi* he does not profess to understand, but the fact that it gives relief he has often observed.—ED. REC.]

**Herpes Circinatus, or Ring-worm.**—An obstinate case of the above (Eclectic Medical Journal), of eight months' standing, was cured by the following:

℞ Fowler's solution of arsenic...........ʒ ss.

Phytolacca...............................℥ ii.

Water.....................................℥ iv. M.

Dose, a teaspoonful four times a day. In addition to the above treatment, gave for external application:

℞ Citrine ointment.....................one part.
Simple cerate.....................three parts.

**Bleeding Hemorrhoids.**—Dr. J. C. Peters (New York Medical Journal) says he has used the tincture of hamamelis internally. He preferred a tincture made of the plant and leaves. One patient, suffering from bleeding hemorrhoids for six or eight years, was cured with it after other remedies proved valueless. The patient was a lady, who would not allow any examination. He gave from ʒj. to ʒiij. of the tincture three times a day.

**Bromo-Hydrate of Quinine as an Antipyretic.**—Esquerdo and Cahis (Independ. Med. and Courrier Med.) found this salt reduce the temperature in typhoid fever four to six degrees Fahr., given in doses of fifty to seventy centigrammes (seven to ten grains.) It has also been used in phthisis.—*The Doctor.*

**Hair-Dyes.**—A dark-brown color may be imparted to hair by various methods. The following are among the best, and are altogether harmless:

1. One part of pyrogallic acid is dissolved in a mixture of two parts of eau de cologne and 38 parts of distilled water. The hair having been freed from fatty matters by luke-warm soap-suds, it is well moistened with the above solution every other day, and, when dry, faintly annointed with hair-oil.

2. 10 parts of fresh walnut-leaves (*juglans regia*), gathered in July, are digested for two days, under frequent stirring, with twenty parts of alcohol and 20 of water. The liquid is removed and expressed, filtered, and concentrated to a syrup over a gentle heat. 10 parts of the resulting brownish-black syrup are diluted with 15 parts of alcohol and 5 parts of water, and perfumed according to fancy. The hair having been freed from fat in the usual manner, it is moistened with the dye, brushed well with a soft brush, and afterwards oiled.

**Burns.**—Take 440 grammes (6790 troy grains) of very clear glue broken into small pieces, and soften it in a litre (2 pints 14½ fluid drachms, apoth.) of cold water; then place in a water-bath and dissolve the glue, to which add 60 grammes (926 troy grains) of glycerine, and 22 grammes (339½ tr. grs.) of phenic acid. Continue the evaporation until a brilliant pellicle is formed upon the surface of the mixture. Upon cooling, this forms an elastic mass, which must be liquefied by heat whenever it is desired to use it. It is to be applied by means of a large hair pencil, and in less than two minutes a brilliant, flexible and almost transparent layer is obtained.—*Apothek. Zeitung.*

**For Dyspepsia.**—Dr. Jas. Case, of Georgia, writes:

"Having seen in the last number of your excellent journal, page 35, a prescription for dyspepsia, I would like to say that I have for many years used with wonderful success, in dyspepsia, the following prescription:

| | | |
|---|---|---|
| ℞ | Acid hydro-cyan., dil....... | 49 drops. |
| | Bismuth sub-nit............ | 100 grs. |
| | Tinct. cascarilla............ | 1 oz. |
| | Infus. " ............ | 15 ". |

M. S. Shake well.

"Take one tablespoonful three times daily, before each meal."

**Pulmonary Alterative Pills.**—Dr. Q. C. Smith, in *Pac. Med. and Surg. Journal*, recommends:

| | | |
|---|---|---|
| ℞ | Hypophosphite of quinia...... | 16 grains |
| | Iodide of arsenic............. | 1 " |
| | Sulphide of lime.............. | 16 " |
| | Ext. of nux vomica........... | 2 " |

Mix. Make sixteen pills. To be sugar-coated.

One pill to be taken three times a day.

**Spirits of Nitre Poisonous.**—A case of fatal poisoning is reported in a child two years old from drinking three ounces of the sweet spirits of nitre. Symptoms: vomiting, followed by dilated pupils, collapse, stertorous breathing, insensibility and death.

**Gonorrhœa.**—Bauer's method of treating gonorrhœa—based upon the theory that it is purely a local disease, the protecting layer of epithelium being thrown off, and the epithelial cells converted into pus cells and discharged, leaving the mucous membrane exposed—is simply the use of a bland injection, which is followed by immediate relief to the pain, and usually results in a cure in about six days as follows.

℞ Inf. flax seed................ ℥ vj.

Watery ext. opium.......... gtts. xviij. M.

To be injected warm every three hours and retained for a few minutes.

**Alopecia or Baldness.**—So called from the Greek word *alopex*, a fox, which is said to be subject to the disease. When the hair falls out as a result of disease the following is recommended as a good application :

℞ Glycerine......................... ʒ j.

Tinct. cantharidis.................. ʒ ij.

Cologne water ..................... ℥ v.

To be applied at night.

As an ointment, use Dupuytren's Pommade, the basis of which is acetate of lead with tinct. cantharidis.

**Hawk-Weed.**—This is a species of *Hieracium*, nat. fam. Compositæ. That which most generally bears this name, and the only one which appears to be used in domestic practice, is *Hieracium venosum* L., also called *Rattlesnake-weed*, *Stripped Blood-wort*, *Veinyleaved Hawk-weed*. Both the roots and the leaves are used. The juice of the fresh leaves is said to be efficacious for removing warts. The powdered leaves and root have been used as a snuff, combined with powdered bloodroot, in polypus of the nose.—*U. S. Dispens.*

**Cough Mixture.**—In any severe cough, where the tongue is red or the throat sore, the following is recommended by Dr. Powell, of London :

℞ Potassii chloratis................ gr. xl.

Morph. muriatis................. grs.ij.

Glycerinæ ....................... ℥ ss.

Syrupii .......................... ℥ iijss. M

Dose one teaspoonful.

**Lactopeptine.**—

℞ Pepsin.................... 8 ounces.
Pancreatine.............. 6 ounces.
Veget. ptyalin or diastase 4 drachms.
Lactic acid .............. 5 fl. drachms.
Hydrochloric acid ....... 5 fl. "
Sugar of mik............40 ounces. M

**Syrup of Chloral.**—

℞ Chloral....................... 80 grains

Distilled water............ 249.6 minims.

Simple syrup, q. s. ad..... 499.2 "

A United States fluid drachm, or sixty minims of this would contain 9.6 grains of chloral.

**For Frosted Feet and Hands.**—

| | | | | |
|---|---|---|---|---|
| ℞ | Almond oil.............. | ℥ ss....(16 | 00) |
| | Beeswax................ | ʒ ss....( 2 | 00) |
| | Spermaceti.............. | ʒ i.....( 4 | 00) |
| | Peruvian balsam........ | ℥ ss....(16 | 00) |
| | Conc. muriatic acid..... | gtt. xlv.( 2 | 70) |

**Paresi's Hæmostatic Collodion.**—

| | | |
|---|---|---|
| ℞ | Officinal collodion........ | 100.00 gmm. |
| | Carbolic acid (pure)....... | 10.00 " |
| | Tannic acid.............. | 5.00 " |
| | Benzoic acid............. | 3.00 " |

M. secund. art.

**Tinctura Saponis Viridis.**—This preparation, which is used in some of the public dispensaries of New York City, as an application in certain skin diseases, is made after the following formula :

| | | |
|---|---|---|
| ℞ | Green soap....................... | 2 ℥. |
| | Alcohol.......................... | 4 fl. ℥. M. |

**Tinctura Saponis Viridis Co.**, used for similar purposes, contains :

| | | |
|---|---|---|
| ℞ | Green soap.................... | 2 ℥. |
| | Alcohol. | |
| | Oil of cade................. | aa 1 fl ℥. |

**Spermatorrhœa.**—

| | | |
|---|---|---|
| ℞ | Bromide sodium........... | 1 ounce. |
| | Fl. ext. ergot.............. | 1½ ounce. |
| | Camphor water........... | 4 ounces. M. |

Dose two teaspoonsful at bed time.

**Esmarch's Treatment of Cancer.**—Fowler's solution, one drop three times a day for three days, then increase the dose by one drop every three days till intolerance of the remedy follows. Apply the following locally :

| | |
|---|---|
| Acid arsenious and morphia muriate, each.... | 0,250 gmm. |
| Calomel.................................... | 2,00 " |
| Powdered gum arabic........................ | 12,00 " M. |

At first sprinkle only a little upon the ulcer, gradually increasing the quantity to a teaspoonful. This overcomes the odor, causes a hard eschar to form, and healthy granulations take place.—*Mich. Med. News.*

**Shock.**—In the Va. Med. Monthly, Dr. A. McGuire recommends quinine to cinchonism to prevent shock from severe surgical operations. A gentleman to whom he had given gr. xv. of quinine the day of the injury suffered amputation two hours after the injury without showing any shock.—*Tol. Med. Journ.*

**Peach Leaves as an Anti-Emetic.**—

| | | |
|---|---|---|
| ℞ | Peach leaves............ | a handful. |
| | Boiling water........... | 1 pint. |

Cool and strain. Give one teaspoonful every half hour.

We have seen violent and persistent vomiting relieved by this remedy.

